# The Combined Use of Hydroxymethylbutyrate and Branched-Chain Amino Acids to Counteract Uremic Sarcopenia

**DOI:** 10.3390/nu18030483

**Published:** 2026-02-01

**Authors:** Giulia Marrone, Manuela Di Lauro, Kevin Cornali, Sabri Shamsan Hassan, Gabriele D’Urso, Luca Di Marco, Sara Dominijanni, Roberto Palumbo, Anna Paola Mitterhofer, Annalisa Noce

**Affiliations:** 1 Department of Systems Medicine, University of Rome Tor Vergata, 00133 Rome, Italymanuela.dilauro@ptvonline.it (M.D.L.); annapaola.mitter@uniroma2.it (A.P.M.); 2 Department of Experimental Medicine, PhD School in Biochemistry and Molecular Biology, University of Rome Tor Vergata, 00133 Rome, Italy; kevin.cornali@students.uniroma2.eu; 3 Nephrology and Dialysis Unit, Nuova Clinica Annunziatella, 00147 Rome, Italy; sabri.hassan56@yahoo.it; 4 UOSD Nephrology and Dialysis, Policlinico Tor Vergata, 00133 Rome, Italy; gabriele.durso@ptvonline.it; 5 National PhD School in Kinesiology and Sport Sciences, University of Verona, Chiostro San Francesco, 37123 Verona, Italy; luca.dimarco@univr.it; 6 Nephrology and Dialysis Unit, ASL Roma 2, St Eugenio Hospital, 00144 Rome, Italy; sara.dominijanni@gmail.com (S.D.); palumbo.dr@gmail.com (R.P.)

**Keywords:** uremic sarcopenia, hemodialysis, food for special medical purposes, hydroxymethylbutyrate, branched chain amino acids, quality of life

## Abstract

Background: Hemodialysis (HD) patients frequently develop muscle wasting and chronic inflammation, conditions associated with functional decline and reduced quality of life (QoL). Nutritional strategies that provide targeted anabolic support without increasing nitrogen load may offer clinical benefits. The aim of this study was to evaluate the possible impact of a food for special medical purposes (FFSMP), composed of free-form branched-chain amino acids, β-hydroxy-β-methylbutyrate, and zinc, on muscle mass and strength, laboratory parameters, physical performance (PP), and QoL in HD patients. Methods: in this randomized double-blind crossover study, 24 adult HD patients received the FFSMP (10 g/day; two sachets) supplementation or placebo for 12 weeks, separated by an 8-week wash-out (protocol code RS 29.23). Measured outcomes included quadriceps rectus femoris thickness (QRFT) muscle, body composition analysis, inflammatory markers, oxidative stress indices, other routine biochemical parameters, PP, and QoL (SF-36 questionnaire). Results: FFSMP supplementation resulted in significant increases in QRFT and in fat-free mass percentage. Reductions in oxidative stress and inflammatory biomarkers were observed. Routine biochemical parameters remained stable, with the exception of a decrease in pre-dialysis urea. Functional performance measures did not differ between treatment periods. Improvements were noted in selected SF-36 domains, specifically energy/fatigue and general health. No major adverse events occurred during the study. Conclusions: In HD patients, this FFSMP produced favorable changes in markers of muscle mass and systemic inflammation without affecting short-term physical performance. These findings support the potential clinical utility of targeted amino acid supplementation in this patient population, highlighting the need for larger, longer-term trials.

## 1. Introduction

Skeletal muscle, composed mainly of water and proteins, is one of the most abundant tissues in the human body and serves as a key indicator of overall protein balance [1]. Commonly, sarcopenia refers to the natural, age-related decline in muscle strength, muscle mass, and overall physical ability. This condition is a key factor behind muscular weakness and impaired mobility, and its negative consequences lead to diminished functional abilities, reduced independence, and a lower quality of life (QoL). Moreover, sarcopenia is strongly linked to increased disability and a higher risk of illness among older adults [2].

Secondary sarcopenia is commonly associated with chronic non-communicable conditions, including cancer, chronic kidney disease (CKD), and diabetes mellitus. Among individuals with CKD, uremic sarcopenia (US) is one of the most prevalent complications, particularly in patients with end-stage kidney disease (ESKD) [3,4,5]. This condition is linked to poorer quality of life as well as a higher risk of disability and mortality [6,7]. Its etiology is multifactorial and still not completely clarified; it includes chronic low-grade inflammation, episodic catabolic states, loss of nutrients through dialysis, metabolic acidosis, and hormonal disturbances, all of which may promote its development and progression [8]. Persistent inflammation can blunt protein synthesis even in the presence of adequate energy and protein intake [8]. Moreover, metabolic acidosis—frequently observed in advanced CKD—has been shown to enhance protein breakdown via activation of the ubiquitin–proteasome pathway (UPP). Additional alterations in CKD patients include reduced muscle protein synthesis and mitochondrial function, structural and metabolic abnormalities of muscle fibers, and increased arterial stiffness [9,10,11,12,13,14,15,16,17].

Mitochondrial oxidative stress, which reflects impaired mitochondrial function in muscle, triggers proteolytic signaling pathways that favor muscle wasting [18,19] and may contribute to cardiovascular diseases in CKD.

The most common anomalies observed in CKD muscle biopsies are the specific atrophy of type II muscle fibers [20,21] and reduced protein synthesis. These conditions induce a negative nitrogen balance [22], inexorably impoverishing the skeletal muscle mass [23,24], and thus contributing to US onset. Moreover, US represents a central element of the frailty syndrome and is also a powerful predictor of disability, morbidity, and mortality in CKD patients. In addition, US may further challenge the sustainability of health and social care systems, which are increasingly burdened by a growing elderly population [2,25,26,27,28,29].

While increasing lifespan is an important public health goal, it is equally crucial that extended years are lived with independence. Therefore, identifying cost-effective interventions to prevent frailty represents a key public health challenge in CKD.

The assessment of intermuscular adipose tissue (IMAT) also plays a relevant role and has been linked to multiple metabolic, muscle-related, and mobility dysfunctions prevalent in CKD [30,31,32,33,34,35,36,37,38,39].

Moreover, hemodialysis (HD) patients are characterized by insulin resistance, metabolic acidosis, chronic microinflammation, malnutrition, oxidative stress and cardiovascular disease, conditions that activate the ATP-dependent ubiquitin–proteasome system (UPS) [40,41,42,43], which drives muscle wasting due to amino acid/protein loss [44,45]. HD sessions are associated with substantial losses of proteins and amino acids, thereby increasing the risk of malnutrition. To counterbalance these losses and the associated inflammatory stress, a higher dietary protein intake is typically recommended to support muscle maintenance [46]. In fact, in addition to pharmacological intervention [47], adjuvant non-pharmacological therapies to prevent these poor outcomes in US patients could be represented by protein-controlled dietary nutritional therapy (DNT) (>1.1/1.2 g of proteins/kg of body weight/day in HD) [48,49], a kind of food for special medical purposes (FFSMP) based on branched-chain amino acids (BCAAs) and a program of adapted physical activity (APA) [50]. Specifically, BCAAs are ergogenic supplements, primarily containing leucine and its metabolite β-hydroxy-β-methylbutyrate (HMB) [51,52,53]. Previous investigations have indicated that this fast-acting free-form essential amino acid supplements (FFSMPs) are associated with improvements in muscle strength, muscle mass, and gait speed [54], and may also play a protective role against the development of sarcopenia in older adults, mitigating muscle protein catabolism [55,56,57,58].

The primary objective of this randomized, double-blind crossover study was to investigate the effects of an FFSMP (based on BCAAs and HMB) on muscle mass, strength, and physical performance (PP) in a cohort of hemodialysis patients over a three-month period, at a daily dose of 10 g, compared with placebo. The secondary objective was to evaluate the impact of FFSMP supplementation on patients’ QoL.

## 2. Materials and Methods

### 2.1. Patients

Twenty-four ESKD patients (17 males and 7 females) undergoing HD, referred at the UOSD of Nephrology and Dialysis of the Fondazione Policlinico Tor Vergata (Rome, Italy), the Dialysis Centre of Nuova Clinica Annunziatella (Rome, Italy) and the UOC Nephrology and Dialysis of the Sant’Eugenio Hospital (Rome, Italy), were enrolled.

Inclusion criteria were as follows:

- Age between 45 and 80 years;
- Both sexes;
- Signature and acceptance of informed consent;
- ESKD under HD treatment for at least three months;
- Utilization of arteriovenous fistula as vascular access for HD.

Exclusion criteria were as follows:

- Presence of solid or hematological malignancies;
- HIV, HbsAg+, HCV+ patients;
- Non-acceptance of informed consent;
- Subjects with inflammatory and/or infectious diseases in the acute phase;
- Pregnancy and lactation;
- Intake of FFSMP based on essential amino acids in the last three months;
- Intake of FFSMP based on antioxidants in the last three months.

Prior to study initiation, all enrolled participants signed an informed consent. The study protocol was conducted in accordance with the principles of the 1975 Declaration of Helsinki and received approval from the Independent Ethics Committee of the Fondazione Policlinico Tor Vergata (protocol code RS 29.23).

**Figure 1:**
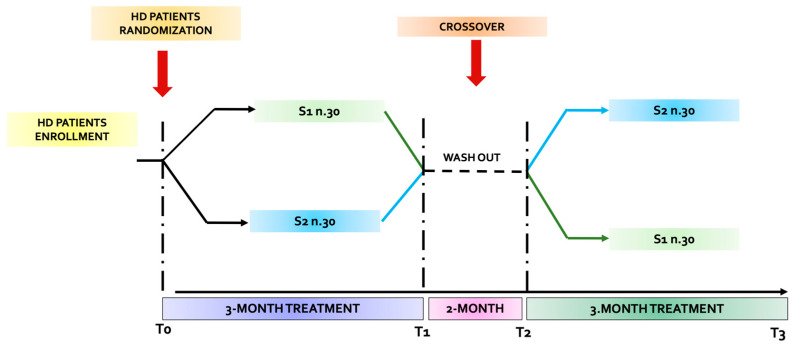
Flow chart of the study.

The study was a randomized double-blind crossover study. Patients were divided into two homogeneous groups: S1 and S2 (Figure 1). In both groups, only the timing of treatment was indicated. The allocation procedure was implemented to assign participants to the FFSMP and placebo treatment. The sequence of exposure (FFSMP–placebo or placebo–FFSMP) was determined using a computer-generated randomization schedule to ensure unbiased allocation and to minimize potential order effects. A wash-out period of 2 weeks was incorporated between the two phases to prevent carryover effects.

Four observation times were outlined in the protocol:

- T_0_: enrollment time;
- T_1_: 3 months from the start of the study;
- T_2_: after the 2-month wash-out period (i.e., 5 months after the start of the study);
- T_3_: a further 3 months after the wash-out period (i.e., 8 months after the start of the study).

The patients of the S1 group took a daily dose of 10 g of highly bioavailable essential amino acids (nr. 2 sachets/day) dissolved in 100 mL of water on an empty stomach for 3 months. The second subgroup (S2) took placebo (PLA) (n.2 sachets/day), for 3 months. After a 2-month wash-out, the group (S1) that consumed the FFSMP consumed PLA in the same manner for the next 3 months, while the group (S2) that initially consumed PLA consumed the FFSMP, in the same manner as described above, for the further 3 months.

At the enrollment time (T_0_), all patients underwent a detailed medical examination, including recent and remote pathophysiological anamnesis, and at each observation time, all patients underwent a physical examination, an evaluation of laboratory, inflammation and oxidative stress parameters, an assessment of anthropometric parameters and body composition, measurement of quadriceps rectus femoris thickness (QRFT), ultrasound examination, measurement of muscle strength and PP, and the administration of a questionnaire on perceived QoL.

### 2.2. Composition of the Food for Special Medical Purposes and Placebo

The FFSMP tested was a mixture of BCAAs—L-leucine, L-isoleucine, and L-valine in a 2:1:1 ratio—combined with HMB, a leucine-derived metabolite. Each 5 g sachet provides L-leucine 1250 mg, L-isoleucine 625 mg, L-valine 625 mg, HMB 750 mg, zinc 5 mg, and 1.3 g of carbohydrates. The product contains no fats, proteins, gluten, or lactose and yields 13 kcal when dissolved in 100 mL of water.

The placebo used in this study was formulated to be visually and organoleptically identical to the FFSMP. It consisted solely of maltodextrin at a concentration of 3255 mg *per* dosage form (df). The placebo was designed to match the appearance, texture, and packaging of the active intervention, while lacking any pharmacologically active ingredients.

### 2.3. Laboratory Parameters, Biomarkers of Inflammation and Oxidative Stress

At the study time points, namely at baseline (T_0_) and at each other protocol observational time (T_1_, T_2_, T_3_), laboratory parameters and biomarkers of inflammation and oxidative stress were monitored. In particular, we evaluated biomarkers of renal function, electrolytes, lipid profile, iron profile, complete blood count, blood glucose, uric acid, parathyroid hormone (PTH), and vitamin D. Moreover, inflammatory parameters such as C-reactive protein (CRP), erythrocyte sedimentation rate (ESR), and inflammatory cytokines, such as interleukin-6 (IL-6), were evaluated.

All laboratory analyses were performed using the Dimension Vista 1500 system (Siemens Healthcare Diagnostics, Milan, Italy), with the exception of the lipid profile, which was measured through standard enzymatic colorimetric methods (Roche Modular P800, Roche Diagnostics, Indianapolis, IN, USA).

All assays followed established protocols in the clinical chemistry laboratories of the Fondazione Policlinico Tor Vergata. For the evaluation of oxidative stress and antioxidant defenses, capillary blood samples were collected from all participants and analyzed using the CR4000 photometer, employing the free oxygen radicals test (FORT) and the free oxygen radicals defense (FORD) assay. These colorimetric assays use different solution-catalyzed reactions to quantify the levels of reactive oxygen species and antioxidant molecules [59].

### 2.4. Measurement of Anthropometric Parameters and Body Composition Assessment

Anthropometric measurements of the enrolled patients, including body weight (kg) and height (m), were obtained using a scale (seca GmbH & Co. KG, model 700, Hamburg, Germany) equipped with an integrated stadiometer. Body weight was registered with a precision of 0.01 kg and height with a precision of 0.1 cm. Body mass index (BMI) was subsequently calculated as weight divided by height squared (kg/m^2^).

Body composition was evaluated in all HD patients using bioelectrical impedance analysis (BIA) [60,61] (EGF Plus^®^ software Bodygram HBO, Estor, Pero, MI, Italy).

During the BIA measurements, participants lay in a supine position on a non-conductive surface, having removed their shoes and socks. Two sets of electrodes were applied to dry skin on the same side of the body, carefully avoiding the limb with an arteriovenous fistula. The BIA assessment included the following parameters: resistance (Rz, Ω), reactance (Xc, Ω), phase angle (PhA, °), total body water (TBW, %), intracellular water (ICW, %), extracellular water (ECW, %), fat mass (FM, %), fat-free mass (FFM, %), body cell mass (BCM, %), and basal metabolic rate (BMR, kcal/day).

To overcome the potential bias due to fluid retention, induced by the oligo-anuric condition of HD patients, BIA was performed at the end of dialytic session in order to reach the dry weight, corresponding to the post-dialysis normohydrated state, after the long interdialytic interval. This approach minimized the confounding effect of extracellular water shifts and ensured that impedance-derived parameters accurately reflected changes in BCM%, FM%, and FFM% rather than the variations in hydration status [62].

### 2.5. Ultrasonographic Evaluation of Muscle Mass

At the scheduled study evaluations—baseline (T_0_) and subsequent protocol time points (T_1_, T_2_, T_3_)—all participants underwent ultrasound assessment of QRFT at the 1/2 and 2/3 sites. This examination constitutes a novel diagnostic approach that is effective for identifying muscle mass loss in patients with CKD [63,64,65,66,67]. Ultrasound assessments were performed using an Esaote MyLab70 XVision system (Genoa, Italy) equipped with an LA523 linear probe, operating in B-mode with a 7.5 MHz transducer. All examinations were conducted by the same operator (A.N.).

Quadriceps rectus femoris thickness (QRFT) was assessed at the 1/2 and 2/3 points by performing three bilateral measurements with patients in a supine position and knees fully extended. The measurements were conducted at two anatomical landmarks: the midpoint between the anterior superior iliac spine and the superior border of the patella (QRFT 1/2), and the junction between the distal third and proximal two-thirds of the quadriceps muscle (QRFT 2/3). The ultrasound probe was aligned perpendicular to the muscle’s longitudinal axis, using a generous layer of gel and applying minimal pressure to prevent compression of the underlying tissue [65,67].

### 2.6. Evaluation of Muscle Strength and Physical Performance

Functional assessments were carried out at baseline (T_0_) and at each protocol observational time (T_1_, T_2_, T_3_).

(a) Muscle strength was assessed using the hand grip strength (HGS) test, which employs a dynamometer to measure isometric handgrip force (Jamar Plus, Performance Health, Warrenville, IL, USA). The patients were seated and were instructed to grip the dynamometer as forcefully as possible using the arm, without the vascular access for hemodialysis (arteriovenous fistula), at 90° close to the hip. The test was performed three times, using the same arm, and the average value was calculated. The standardized cut-offs of the HGS test were <27 kg for men and <16 kg for women [68,69].
(b) Three tests were employed to evaluate PP [70,71]:
  - The short physical performance battery (SPPB) [70] comprises three components: gait speed assessed over a 4 m walk, lower-limb strength evaluated through the five-times sit-to-stand test, and balance measured using the tandem stance. Each component is scored on a scale of 0 to 4, and the total score reflects overall PP. A maximum score of 12 denotes optimal performance.
  - The stair climb power test (SCPT) [72] evaluates lower-limb power. Patients were instructed to climb 10 steps as quickly as possible, without running or jumping, and the time required to complete the task was recorded.
  - Six-minute walk test (SMWT) [71,73] evaluates functional capacity. Patients walked for six minutes along a 30 m flat corridor, without running. Total distance covered and rate of perceived exertion were recorded at the end of the test using the Borg CR10 Scale [74].

### 2.7. Questionnaires

During the study, three questionnaires, “PREvención con DIeta MEDiterránea” (PREDIMED), the “36-Item Short-Form Health Survey” (SF-36) and the “International Physical Activity Questionnaire” (IPAQ) were administered to evaluate, respectively, the adherence to the Mediterranean Diet (MD), perceived patient’s QoL and the physical activity level.

The PREDIMED questionnaire was used to exclude possible lifestyle-related biases. This questionnaire consists of 14 items, each valued at 1 point, for a total possible score of 14. Based on the final score, participants were categorized into three levels of adherence to the MD: low adherence (≤5 points), moderate adherence (6–9 points), and high adherence (≥10 points) [75].

The SF-36 is a self-administered questionnaire designed to evaluate patients’ perceived QoL. It assesses eight domains: physical functioning, role limitations due to physical health, role limitations due to emotional problems, energy/fatigue, emotional well-being, social functioning, pain, general health, and health change [76].

The IPAQ is a widely used instrument, designed as a standardized self-report questionnaire for physical activity surveillance. IPAQ can provide comparable data on health–related physical activity and sedentary behavior [77,78,79,80,81].

### 2.8. Statistical Analysis

Statistical analyses were performed by first organizing all data in an Excel spreadsheet (Microsoft, Redmond, WA, USA), followed by processing with IBM SPSS Statistics for Windows, version 25.0 (Chicago, IL, USA). Continuous variables, confirmed to be normally distributed using histograms and the Kolmogorov–Smirnov test, were expressed as mean ± standard deviation. One-way ANOVA was used to assess the uniformity of epidemiological and anthropometric characteristics among participants. Inferential testing employed parametric methods (e.g., Student’s *t*-test, Fisher’s F test) for quantitative variables and non-parametric approaches (e.g., Mann–Whitney U test, Fisher’s exact test) for qualitative variables. The non-parametric median test was applied to categorical data. Comparisons focused on differences between the FFSMP supplementation period and the placebo period, with statistical significance defined as *p* ≤ 0.05.

### 2.9. Power Analysis

A total of 24 patients undergoing dialysis therapy were included in the study. They were randomly assigned, using block randomization, into two balanced subgroups of 12 patients each (S1 and S2). Subgroup S1 received 10 g/day of the FFSMP for 3 months, while subgroup S2 received the placebo for the same duration. After a 2-month wash-out period, the treatments were crossed over: S1 received the placebo and S2 received FFSMP for an additional 3 months.

The choice of 24 patients was based on the expected statistical power of the study. Using a two-tailed test with α = 0.05 and β = 0.10 (power = 90%), this sample size allows the study to detect a moderate difference between FFSMP and placebo when comparing the same patients before and after the crossover. Therefore, the calculated power of the study is 0.912.

## 3. Results

**Figure 2:**
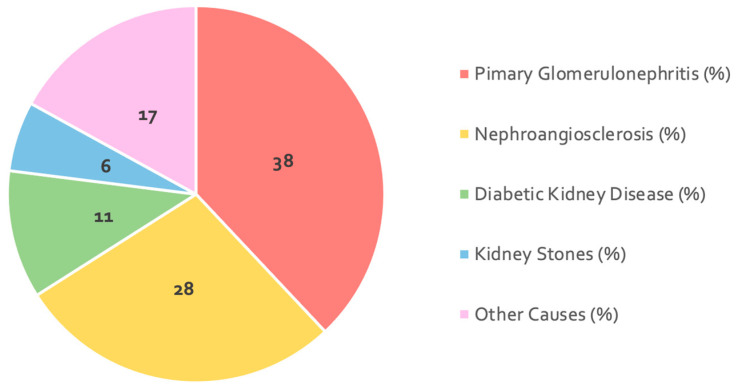
The prevalence of ESKD etiology in the study population (expressed in percentage).

The epidemiological characteristics and comorbidities of the enrolled patients are summarized in Table 1. Figure 2 illustrates the prevalence of ESKD etiology in the study population.

During the survey, we did not register any dropout or side effects induced by FFSMP consumption.

Table 2 shows the laboratory parameters of the enrolled patients, monitored at each time-point of the study.

At the end of the period of FFSMP consumption, pre-dialysis azotemia (*p*-value= 0.0007), transferrin (*p*-value = 0.0046), ferritin (*p*-value = 0.0381), and zinc (*p*-value = 0.0003) showed a statistically significant decrease compared to the placebo period.

The inflammatory and oxidative stress biomarkers, monitored before (PRE-treatment) and after (POST-treatment) the intake of the FFSMP and before (PRE-treatment) and after (POST-treatment) the intake of PLA, are reported in Table 3. At the conclusion of the study, the ESR (*p*-value = 0.0001), the CRP (*p*-value = 0.0311) and the IL-6 (*p*-value = 0.0412) showed a statistically significant decrease after 12 weeks of treatment with the FFSMP compared to the PLA period.

Regarding the evaluation of oxidative stress, the FORT (*p*-value = 0.0159) and the FORD test (*p*-value = 0.0385) were statistically reduced at the end of the 12-week intake period of the FFSMP compared to the PLA period.

The results of the QRFT, reported in Table 4, demonstrate a statistically significant increase in the quadriceps femoris muscle at the proximal and distal landmarks, assessed before (PRE-treatment) and after (POST-treatment) the intake of the FFSMP and before (PRE-treatment) and after (POST-treatment) the intake of the PLA. In detail, the ultrasonographic examination of the left proximal landmark (*p*-value = 0.0017), of the right proximal landmark (*p*-value = 0.0010), of the left distal landmark (*p*-value = 0.0355), and of the right distal landmark (*p*-value = 0.0011) showed a statistically significant increase in the FFSMP period compared to the PLA period.

Table 5 shows the parameters obtained by BIA, monitored before (PRE-treatment) and after (POST-treatment) the intake of FFSMP and before (PRE-treatment) and after (POST-treatment) the intake of PLA. At the end of the FFSMP consumption period, the FFM% (*p*-value = 0.0464) higlighted a significant increase compared to the PLA period. Moreover, we observed a reduction in resistance (*p*-value = 0.0226) and an enhance of phase angle (*p*-value = 0.0187).

Table 6 reports the parameters of PP and muscle strength, monitored before (PRE-treatment) and after (POST-treatment) the intake of FFSMP (PRE-treatment) and before (PRE-treatment) and after (POST-treatment) the intake of the PLA. No statistically significant changes were observed in any of the measured parameters.

Regarding the PREDIMED and the IPAQ, they did not display statistically significant variations, thus eliminating the possible bias induced by lifestyle changes on the results obtained during the study.

**Figure 3:**
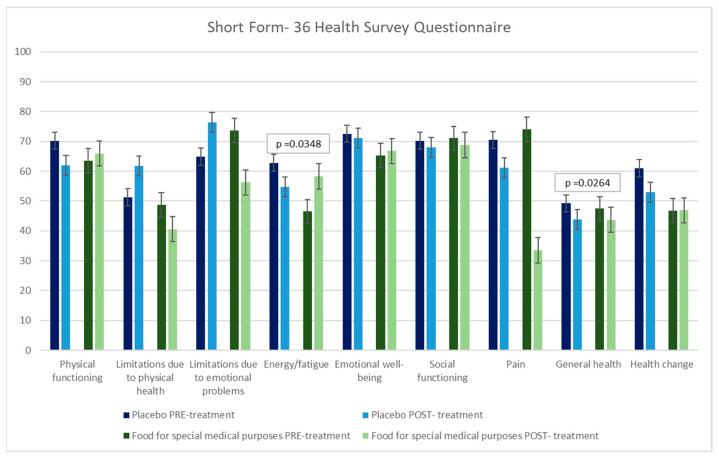
Short form-36 health survey questionnaire administered before (PRE-treatment) and after (POST-treatment) the intake of the food for special medical purposes and before (PRE-treatment) and after (POST-treatment) the intake of the placebo.

Figure 3 shows the results of the SF-36 questionnaire administered to patients during the study. The result obtained after FFSMP consumption period is significant. In particular, we observed an increase in the domains of energy/fatigue (*p*-value = 0.0348) and general health (*p*-value = 0.0264) in the period after taking the FFSMP compared to the PLA.

## 4. Discussion

US represents a deeply debilitating complication in ESKD patients, particularly among those in RRT. In fact, it is associated with an increased risk of falls, fractures, and hospitalization. Both hospitalization and diminished functional independence significantly impact healthcare costs, including the economic burdens on caregivers. Early identification is therefore essential to quickly initiate an appropriate multidisciplinary treatment and improve clinical outcomes [82].

The correction of metabolic acidosis and the implementation of tailored exercise programs accompanied by the nutritional interventions, including the use of FFSMP, are key components of a multidisciplinary approach [83]. In particular, evidence suggests that nutritional interventions targeting the microbiota (such as dietary modifications rich in fiber, probiotics, and synbiotics) can reduce the production of the uremic toxins and microinflammation [84]. The combined treatment may further enhance the patient’s QoL by supporting muscle mass preservation and functional capacity.

Currently, patients presenting protein-energy-wasting often experience limitations in performing activities of daily living (ADLs), with a consequent reduction in their functional autonomy. Due to the clinical severity of sarcopenia, the implementation of targeted therapeutic strategies in order to slow down progression and preserve muscle function is of fundamental importance [85].

The first study that investigated the effects of BCAAs in ESKD was conducted by Hiroshige et al. in a population of elderly malnourished HD patients. This randomized double-blind crossover study highlighted how oral supplementation with BCAAs counteracts anorexia and low protein and caloric intakes. The improvement in the overall nutritional status of the HD patients was also shown through the significant increase in serum albumin plasma levels [86]. The effects of BCAAs are also reported in the case report of Nogueira-Pérez Á et al. Supplementation with HMB in an HD patient resulted in enhanced muscle mass and strength, as well as an enhancement of insulin-like growth factor-1 [87]. The response to HMB supplementation was also studied in the retrospective analysis conducted by Sipahi et al. on diabetic HD patients. The authors emphasized that the administration of HMB, in combination with essential amino acids, promoted wound healing [88].

The mitigation of US appears achievable by counteracting systemic inflammation through inhibition of the ATP-dependent UPS. Concurrently, oxidative stress contributes to muscle fiber damage and facilitates IMAT infiltration, ultimately leading to the replacement of myocytes with adipocytes [31,89].

In our study, the combination of HMB with anti-inflammatory molecules (namely zinc) produce additional beneficial effects on muscle mass, systemic inflammation and oxidative stress markers, never previously reported in the scientific literature. In fact, the positive response obtained following administration of our FFSMP is partly related to the synergistic effects of zinc with HBM, as well as from the beneficial action of the BCAAs themselves. Collectively, these mechanisms underscore the importance of integrating nutritional strategies with adapted physical activity protocols in the US management.

The findings of our randomized double-blind crossover study indicate that the administration of the FFSMP is associated with a notable increase in muscle mass at both the medial and distal regions of the quadriceps femoris, as assessed via ultrasonographic imaging [90,91,92]. Moreover, patients reported significant improvements in their perceived overall health status and increased levels of energy, as evidenced by the SF-36 questionnaire, demonstrating an improvement in the ADLs performance.

However, functional performance tests did not differ after treatment periods. This result highlights how the FFSMP produced favorable changes in muscle mass and, at the same time, protected against deterioration in PP, as is frequently expected in HD patients.

These observed effects are attributable to the presence of BCAAs (specifically, L-Leucine 2500 mg, L-Isoleucine 1250 mg, L-Valine 1250 mg, and protein content = 0, as the product exclusively contains free-form amino acids) and HBM (1500 mg).

In detail, BCAAs exert potent anabolic effects, mitigating muscle protein degradation and serving as an energy substrate during the physical exercise. Specifically, they enhance skeletal muscle protein synthesis through the activation of the mTOR signaling pathway [93]. Moreover, they attenuate proteolysis via inhibition of the ATP-dependent UPS [94] and reduce cellular oxidative stress [95].

These results are further substantiated by a body composition evaluation. After the FFSMP period, there was a significant reduction in resistance parameter, alongside the increase in FFM% and PhA. The elevation in FFM% suggests an increase in the lean mass, while the increase in PhA serves as a well-established indicator of cellular health and is correlated with favorable clinical outcomes [55]. Collectively, these findings highlight a comprehensive improvement in body composition, including enhanced cellular integrity.

After 12 weeks of intervention, inflammatory markers—such as ESR, CRP and IL-6 —were significantly reduced. This effect may be partially attributed to the anti-inflammatory properties of zinc (10 mg), contained within the FFSMP. Zinc works as a critical modulator of immune response; its deficiency triggers inflammatory pathways by promoting the synthesis of pro-inflammatory cytokines [96]. Several studies have confirmed an inverse correlation between zinc levels and circulating pro-inflammatory cytokines, reinforcing its anti-inflammatory role [97].

According to these observations, oxidative stress assessments, via FORD and FORT testing, revealed a reduction in FORT values following the administration of the FFSMP, while the increased FORD values reflected the strengthened of endogenous antioxidant defenses.

Additional evidence of the patient’s improved metabolic status was provided by the significant reduction in pre-dialysis blood urea nitrogen, which suggests a shift toward a reduced catabolic state and reduced urea production. These parameters show an enhancement in overall metabolic homeostasis.

The primary study limitations are the small sample size and the lack of monitoring of physical exercise performed by patients and the BCAA/HMB metabolite concentrations during the study.

Other limitations are that we did not apply adjustments for the large number of comparisons performed and the short time of observation. The latter explains the absence of statistically significant changes in PP outcomes [50,98,99].

## 5. Conclusions

In conclusion, the present study provides interesting evidence that the FFSMP, containing BCAAs and HMB, effectively counteracts US in HD patients. Its consumption not only promotes an increase in muscle mass and strength but also seems to improve the patients’ QoL, as reflected in SF-36 scores. Moreover, we also observed a significant reduction in both systemic inflammation and oxidative stress. These findings support the potential utility of this FFSMP as a valuable adjuvant therapy for the management of US in patients undergoing RRT, offering a multidimensional approach to improving both physiological and clinical outcomes.

## Figures and Tables

**Table 1:** Epidemiological characteristics and comorbidities of the enrolled patients.

number	24
Age (years)	61.5 ± 12.05
Males: females	17:7
Dialysis age (months)	123.3 ± 104.3
Comorbidities (%):	
Arterial hypertension	81
Diabetes mellitus	4.7
Dysthyroidism	19
AMI	24
AA ectasia or aneurysm	9.5
Dyslipidemia	19
COPD	9.5
OSAS	9.5
Cerebral ischemia	14.2

Values expressed as mean ± standard deviation. Abbreviations: AMI, acute myocardial infarction; COPD, chronic obstructive pulmonary disease; OSAS, obstructive sleep apnea syndrome.

**Table 2:** Laboratory parameters monitored before (PRE-treatment) and after (POST-treatment) the intake of the food for special medical purposes and before (PRE-treatment) and after (POST-treatment) the intake of the placebo.

	Food for Special Medical Purposes			Placebo		
Parameters	PRE-Treatment	POST-Treatment	*p*-Value	PRE-Treatment	POST-Treatment	*p*-Value
Red blood cells (millions/μL)	3.6 ± 0.6	3.5 ± 0.5	0.9712	3.6 ± 0.60	3.3 ± 0.90	0.8623
Hemoglobin (g/dL)	11.1 ± 1.1	11.4 ± 1.2	0.7635	11 ± 1.44	11 ± 0.76	0.6534
White blood cells (thousands/μL)	7.2 ± 2.0	6.3 ± 2.5	0.8132	6.8 ± 1.89	6.3 ± 2.63	0.8821
Platelets (thousands/μL)	201.0 ± 62.8	199.5 ± 55.5	0.9821	207.6 ± 62.77	208.4 ± 2.44	0.9945
Neutrophils (%)	70.3 ± 12.2	69.5 ± 7.5	0.9215	70.3 ± 9.18	68.8 ± 7.59	0.7211
Monocytes (%)	7.4 ± 2.6	7.3 ± 2.8	0.9112	7.3 ± 2.57	7.8 ± 2.10	0.8871
Eosinophils (%)	3.1 ± 0.9	3.4 ± 0.3	0.8712	2.8 ± 1.76	3.2 ± 1.32	0.8653
Basophils (%)	0.6 ± 0.3	1.0 ± 1.0	0.4732	0.6 ± 0.40	0.6 ± 0.36	0.9534
Lymphocytes (%)	17.1 ± 6.0	19.3 ± 5.6	0.5645	18.9 ± 6.35	19.7 ± 6.71	0.9534
Pre-dialysis azotemia (mg/dL)	165.6 ± 29.3	136.7 ± 31.9	**0.0007**	140.6 ± 36.02	137.5 ± 36.98	0.5532
Transferrin (mg/dL)	178.1 ± 36.2	159.7 ± 27.2	**0.0046**	160.4 ± 26.49	171.6 ± 39.18	0.6425
Ferritin (ng/mL)	471.5 ± 250.5	359.2 ± 201.1	**0.0381**	575.90 ± 285.5	659.6 ± 586.6	0.5342
Glicaemia (mg/dL)	105.7 ± 28.5	98.7 ± 49.0	0.5263	131.0 ± 119.19	105.4 ± 30.49	0.2253
Uric acid (mg/dL)	5.9 ± 1.2	5.4 ± 1.2	0.4562	6.2 ± 0.70	6.3 ± 1.28	0.4364
Sodium (mmol/L)	139.6 ± 2.8	138.3 ± 2.2	0.3425	139.4 ± 3.82	138.9 ± 2.41	0.3542
Potassium (mmol/L)	5.1 ± 0.6	5.5 ± 0.5	0.6574	5.2 ± 0.89	5.5 ± 0.65	0.5342
Calcium (mmol/L)	8.8 ± 0.7	8.6 ± 0.6	0.3425	8.7 ± 0.75	8.7 ± 0.43	0.4536
Phosphorus (mmol/L)	5.0 ± 1.3	4.9 ± 1.5	0.7364	5.6 ± 1.64	4.9 ± 1.28	0.9836
Chlorine (mmol/L)	106.2 ± 3.2	108.2 ± 8.3	0.7634	109.5 ± 3.10	105.5 ± 4.07	0.5712
Magnesium (mmol/L)	2.2 ± 0.43	2.3 ± 0.32	0.1892	2.1 ± 0.35	2.3 ± 0.44	0.4990
Zinc (µg/dL)	68 ± 9	89 ± 8	**0.0003**	70 ± 10	72 ± 9	0.7137
Total cholesterol (mg/dL)	133.5 ± 30.9	138.9 ± 35.2	0.9980	141.7 ± 29.34	136.0 ± 29.71	0.7645
HDL cholesterol (mg/dL)	37.5 ± 11.7	37.1 ± 12.4	0.9375	47.5 ± 33.17	38.4 ± 12.04	0.5654
LDL cholesterol (mg/dL)	78.4 ± 30.3	82.4 ± 31.6	0.6748	82.7 ± 26.88	78.2 ± 26.36	0.6726
Triglycerides (mg/dL)	128.3 ± 69.0	146.5 ± 77.7	0.3254	135.7 ± 63.56	140.3 ± 58.69	0.8762
Parathyroid hormone (pg/mL)	432.4 ± 380.4	564.0 ± 525.9	0.2290	432.2 ± 403.24	539.4 ± 536.34	0.5731
Vitamin D (ng/mL)	27.3 ± 17.6	30.6 ± 25.1	0.7920	28.1 ± 20.62	21.1 ± 13.71	0.5423

Values are expressed as mean ± standard deviation. Abbreviations: HDL, high-density lipoprotein; LDL, low-density lipoprotein.

**Table 3:** Inflammatory and oxidative stress biomarkers monitored before (PRE-treatment) and after (POST-treatment) the intake of the food for special medical purposes and before (PRE-treatment) and after (POST-treatment) the intake of the placebo.

	Food for Special Medical Purposes			Placebo		
Parameter	PRE-Treatment	POST-Treatment	*p*-Value	PRE-Treatment	POST-Treatment	*p*-Value
ESR (mm/h)	41.8 ± 28.5	21.6 ± 22.3	**0.0001**	34.4 ± 27.21	43.1 ± 30.6	0.5378
CRP (mg/L)	12.7 ± 17.0	3.6 ± 3.7	**0.0311**	11.1 ± 17.36	5.7 ± 7.81	0.2082
IL-6 (pg/mL)	10.9 ± 5.2	3.8 ± 3.4	**0.0412**	10.4 ± 8.74	20.8 ± 6.9	0.0772
FORT (U)	344.3 ± 88.3	293.8 ± 91.8	**0.0159**	316.7 ± 96.47	383.3 ± 84.46	0.2382
FORD (mmol/L Trolox)	0.83 ± 0.6	1.38 ± 0.7	**0.0385**	1.1 ± 0.54	0.8 ± 0.65	0.9273

Values are expressed as mean ± standard deviation. Abbreviations: ESR, eritrocyte sedimentation rate; CRP, c-reactive protein; IL, interleukin; FORT, free oxygen radicals test; FORD, free oxygen radicals defense.

**Table 4:** Ultrasonographic parameters monitored before (PRE-treatment) and after (POST-treatment) the intake of the food for special medical purposes and before (PRE-treatment) and after (POST-treatment) the intake of the placebo.

	Food for Special Medical Purposes			Placebo		
Parameter	PRE-Treatment	POST-Treatment	*p*-Value	PRE-Treatment	POST-Treatment	*p*-Value
QRFT 1/2 left (cm)	1.23 ± 0.3	1.55 ± 0.3	**0.0017**	1.6 ± 0.38	1.5 ± 0.39	0.8532
QRFT 1/2 right (cm)	1.29 ± 0.4	1.73 ± 0.04	**0.0010**	1.6 ± 0.4	1.5 ± 0.4	0.9521
QRFT 2/3 left (cm)	1.13 ± 0.2	1.35 ± 0.39	**0.0355**	1.3 ± 0.24	1.2 ± 0.31	0.9073
QRFT 2/3 right (cm)	1.09 ± 0.3	1.46 ± 0.3	**0.0001**	1.3 ± 0.27	1.1 ± 0.29	0.7289

Values are expressed as mean ± standard deviation. Abbreviations: QRFT, quadriceps rectus femoris thickness.

**Table 5:** Anthropometric and body composition parameters, assessed by BIA, monitored before (PRE-treatment) and after (POST-treatment) the intake of the food for special medical purposes and before (PRE-treatment) and after (POST-treatment) the intake of the placebo.

	Food for Special Medical Purposes			Placebo		
Parameter	PRE-Treatment	POST-Treatment	*p*-Value	PRE-Treatment	POST-Treatment	*p*-Value
Weight (kg)	75.4 ± 16.1	76.5 ± 16.7	0.9473	75.5 ± 15.23	75.6 ± 15.42	0.9647
BMI (kg/m^2^)	25.8 ± 4.6	26.3 ± 4.8	0.8476	25.7 ± 4.44	25.6 ± 4.32	0.9832
Resistance (Ω)	549.1 ± 116.4	516.1 ± 96.2	**0.0226**	537.7 ± 105.92	531.2 ± 95.2	0.6392
Reactance (Ω)	49.8 ± 12.8	48.3 ± 12.1	0.7832	48.0 ± 15.8	50.3 ± 13.79	0.5725
Phase Angle (°)	4.9 ± 0.7	5.5 ± 1.2	**0.0187**	5.1 ± 1.33	5.4 ± 1.35	0.5241
TBW %	54.5 ± 4.8	55.0 ± 5.0	0.8846	55.0 ± 6.54	54.5 ± 4.68	0.8471
ICW %	49.7 ± 6.1	49.7 ± 6.1	0.9546	48.5 ± 9.27	50.5 ± 8.40	0.7265
ECW %	50.2 ± 6.1	50.3 ± 6.1	0.9632	51.5 ± 9.24	49.7 ± 8.32	0.5536
FM %	27.1 ± 6.2	26.2 ± 8.4	0.6453	28.1 ± 6.29	26.7 ± 5.81	0.3635
FFM %	71.7 ± 6.8	74.8 ± 7.5	**0.0464**	73.0 ± 5.15	71.9 ± 6.73	0.5364
BCM %	48.7 ± 6.3	49.3 ± 6.8	0.8543	47.5 ± 9.96	49.5 ± 8.97	0.6473
BMR (Kcal/day)	1532.2 ± 213.1	1535.8 ± 202.1	0.7642	1499.2 ± 233.74	1553.7 ± 235.34	0.3324

Values are expressed as mean ± standard deviation. Abbreviations: BMI, body mass index; ECW, extracellular water; ICW, intracellular water; FFM, fat-free mass; FM, fat mass; TBW, total body water; BCM, body cell mass; BMR, basal metabolism.

**Table 6:** Physical performance parameters and muscle strength monitored before (PRE-treatment) and after (POST-treatment) the intake of the food for special medical purposes and before (PRE-treatment) and after (POST-treatment) the intake of the placebo.

	Food for Special Medical Purposes			Placebo		
Parameter	PRE-Treatment	POST-Treatment	*p*-Value	PRE-Treatment	POST-Treatment	*p*-Value
SPPB	9.8 ± 3.0	10.4 ± 1.9	0.9836	9.9 ± 2.48	10.1 ± 2.15	0.9374
SMWT (m)	322.0 ± 130.6	374.2 ± 134.5	0.2847	339.4 ± 106.04	321.5 ± 112.92	0.7364
SCPT	12.3 ± 5.0	11.9 ± 4.8	0.5364	13.04 ± 5.04	12.0 ± 6.24	0.6473
HGST (kg)	27.9 ± 7.2	31.2 ± 7.2	0.1093	29.1 ± 7.99	29.4 ± 9.07	0.4532

Values are expressed as mean ± standard deviation. Abbreviations: SPPB, short physical performace battery; SMWT, six-minute walking test; SCPT, stair-climb power test; HGST, handgrip strength test.

## Data Availability

The original contributions presented in this study are included in the article. Further inquiries can be directed to the corresponding author.
